# Dual Roles of Host Zinc Finger Proteins in Viral RNA Regulation: Decay or Stabilization

**DOI:** 10.3390/ijms252011138

**Published:** 2024-10-17

**Authors:** Hyokyoung Lee, Sung-Kyun Park, Junghyun Lim

**Affiliations:** 1 Department of Pharmacy, School of Pharmacy and Institute of New Drug Development, Jeonbuk National University, Jeonju 54896, Republic of Korea; 2 Infectious Disease Research Center, Korea Research Institute of Bioscience and Biotechnology (KRIBB), Daejeon 34141, Republic of Korea; 3 Department of Functional Genomics, KRIBB School of Bioscience, Korea University of Science and Technology (UST), Daejeon 34113, Republic of Korea

**Keywords:** zinc finger protein, ZAP, ZCCHC, viral RNA, oncogenic virus

## Abstract

Host defense mechanisms against viral infections have been extensively studied over the past few decades and continue to be a crucial area of research in understanding human diseases caused by acute and chronic viral infections. Among various host mechanisms, recent findings have revealed that several host RNA-binding proteins play pivotal roles in regulating viral RNA to suppress viral replication and eliminate infection. We have focused on identifying host proteins that function as regulators of viral RNA, specifically targeting viral components without adversely affecting host cells. Interestingly, these proteins exhibit dual roles in either restricting viral infections or promoting viral persistence by interacting with cofactors to either degrade viral genomes or stabilize them. In this review, we discuss RNA-binding zinc finger proteins as viral RNA regulators, classified into two major types: ZCCCH-type and ZCCHC-type. By highlighting the functional diversity of these zinc finger proteins, this review provides insights into their potential as therapeutic targets for the development of novel antiviral therapies.

## 1. Introduction

Viral infections are well known to cause a variety of acute and chronic human diseases. Acute viral infections lead to the rapid onset of symptoms and typically run their course within a short period, often cleared by the immune system. However, when the immune system fails to promptly eliminate certain acute viral infections, disasters like the COVID-19 pandemic can occur. In contrast, chronic viral infections persist over long periods and may result in long-term complications or severe diseases, such as cancer. It is now well established that several viruses, such as human papillomavirus (HPV), hepatitis B and C viruses (HBV/HCV), Epstein–Barr virus (EBV), human T-cell lymphotropic virus type 1 (HTLV-1), human herpesvirus 8 (HHV-8), and Merkel cell polyomavirus (MCPyV), are classified as oncogenic viruses due to their ability to potentially induce cancer [1]. These viruses contribute to cancer by disrupting normal cellular processes, such as DNA repair, cell cycle regulation, and apoptosis, leading to tumor formation. Viral vaccines and antiviral therapies can reduce the risk of virus-associated cancers.

Once viral RNA is detected by host intracellular sensors, antiviral pathways are activated to inhibit viral replication, subsequently triggering innate and adaptive immune responses aimed at eliminating the virus. These responses are mediated by cytokines, particularly interferons (IFNs), which play a crucial role in promoting cytotoxic immune responses [2,3,4]. However, in many cases, it can also be difficult to efficiently eliminate the infected viruses because many viruses evade immune detection by exploiting host factors and enhancing their stability within the host environment. Although antiviral drugs play a crucial role in managing viral infections, they face significant limitations such as resistance, toxicity, limited efficacy in chronic diseases, and narrow spectrum of activity.

Recent studies have revealed that certain ZC3H and ZCCHC superfamily proteins containing zinc finger domains in infected cells play crucial roles in binding viral RNAs, inducing antiviral responses, and regulating their replication. Due to these functions, these proteins are of great interest as potential targets for the development of antiviral therapies. In this context, we focus on the interactions between these zinc finger proteins and viral RNAs known to date and categorize the host zinc finger proteins based on their positive or negative roles in viral replication.

## 2. ZC3HAV1

Zinc Finger CCCH-type Antiviral Protein 1 (ZC3HAV1), also known as Poly ADP-ribose Polymerase-13 (PARP-13) or Zinc Finger Antiviral Protein (ZAP) [5], is an RNA-binding protein that targets both positive and negative single-stranded viral RNAs with four zinc finger domains in the N-terminal by binding to specific RNA sequences, CpG dinucleotides [6]. ZAP, initially recovered through cDNA screening from Moloney murine leukemia virus (MLV)-resistant cells [7], has been primarily recognized for its antiviral functions, including the suppression of viral replication and degradation of viral RNA [8]. Recent studies have expanded ZAP’s roles as not only a direct antiviral restriction factor in viral replication but also a regulator of host cell homeostasis in antiviral IFN response [9].

**Figure 1:**
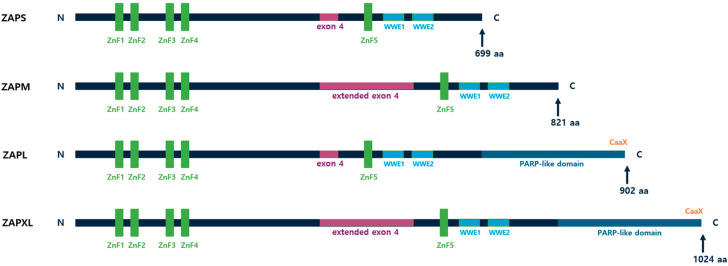
Schematic domain maps of human ZAP isoforms. The N-terminal region contains four key zinc finger domains, with an additional zinc finger domain located centrally, near the WWE1 and WWE2 domains. ZAPL and ZAPXL possess PARP-like domains, which are absent in ZAPS and ZAPM. Additionally, ZAPM and ZAPXL feature an extended exon 4, while ZAPS and ZAPL contain the normal exon 4.

ZAP exists in several isoforms, most notably ZAP-short (ZAPS) and ZAP-long (ZAPL), which are produced from the same gene via alternative splicing [10] (Figure 1). These isoforms share an identical RNA-targeting sequence in their N-terminal regions but differ in their C-terminal domains, which affect their subcellular localization, expression kinetics, and functions [9]. ZAPL, which contains a PARP-like domain with a C-terminal prenylation motif (CaaX) within, acquires hydrophobicity through prenylation by S-farnesyltransferases, leading to the formation of membrane-associated foci that mediate viral RNA degradation in the host cell [11,12]. In contrast, ZAPS, which lacks a PARP-like domain, resides in the cytoplasm and interacts with interferon (IFN) mRNA to maintain cellular homeostasis in antiviral IFN responses by binding preferentially to AU-rich elements (AREs) in the 3′ UTR of IFN mRNAs, promoting their degradation [9]. In addition, another study suggests that ZAPS may also enhance RIG-I signaling by promoting RIG-I oligomerization, thereby stimulating IFN expression [13]. Two other isoforms of ZAP are ZAPM and ZAPXL, which have an extended exon 4 (Figure 1). ZAPXL, like ZAPL, contains a PARP-like domain at the C-terminus, but ZAPM, like ZAPS, lacks this domain. These isoforms exhibit different sensitivities to various types of viruses. It has been suggested that ZAPL and ZAPXL, due to their PARP-like domains, are more sensitive, and have greater antiviral potential against hepatitis B virus (HBV) compared to the other two isoforms [14].

ZAP contains four zinc finger (ZnF) domains in its N-terminal region, grouped into two clusters (ZnF1-2 and ZnF3-4) [10,15], with a fifth ZnF domain (ZnF5) located near two WWE domains in the central region [16,17] (Figure 1). ZnF2 plays a crucial role as a CG-binding pocket, forming hydrogen bonds with CpG dinucleotides in single-stranded RNA, thereby increasing ZAP’s binding affinity [6,15]. ZnF3 functions as a binding pocket for both guanine and cytosine, while ZnF4 serves specifically as a binding pocket for cytosine [6]. Structural studies of the ZnF5-WWE1-WWE2 domains in both mouse and human ZAP have demonstrated that the WWE2 domain exhibits poly (ADP-ribose) binding activity, with the binding site being extended by the groove formed through the WWE1 fold. In contrast to the other zinc finger domains located in the N-terminal region, ZnF5 is thought to facilitate the assembly of these three domains rather than directly engaging in RNA binding [16].

ZAP’s antiviral mechanism involves its selective binding to CpG-rich RNA sequences. In the case of HIV-1, ZAP binding to CpG dinucleotides, but not GpC dinucleotides, inhibits viral replication by recruiting RNA degradation machinery [18]. The optimal ZAP binding motif was proposed as C(n7)G(n)CG, which leads to the recruitment of RNA degradation machinery to inhibit the viral replication [6]. The findings about the number and spacing of CpG dinucleotides, as well as nearby sequences in viral RNA, that influence ZAP sensitivity explain that each CpG dinucleotide had a cumulative antiviral effect, and approximately 15 CpG dinucleotides with adequate spacing, about 14 to 32 nucleotides, were necessary to efficiently inhibit HIV-1 replication [19]. Additionally, the sequences with high UpA near CpG sites (CpG-high and UpA-high) could further enhance the effectiveness of ZAP binding more strongly; however, the number of CpG dinucleotides has a greater impact on binding affinity [20].

The enrichment of uridine (U) or adenosine (A) sequences also enhances the accessibility of ZAP and several cofactors by preventing the formation of stable secondary RNA structures [19]. The cofactors include RNase L, whose cleavage activity gets stronger as the proportion of U and A sequences increases [19,20,21], and co-operation of TRIM25 [22,23] and KHNYN [23], which promote antiviral effects by inducing the production of antibodies against viruses such as HIV-1.

In addition to TRIM25 and KHNYN, several cofactors are involved in ZAP activity (Table 1). RNA degradation activity of ZAP can be triggered through following: (1) interaction of TRIM25 [22,23] and the endoribonuclease KHNYN [23], (2) Riplet co-operating with TRIM25 [24], (3) RNase L activated by OAS3-inducing molecule [20], (4) the complex of the P72 RNA helicase (DDX17) [25] and the DCP1A:DCP2 decapping enzyme [26], (5) the 5′ to 3′ exoribonuclease XRN1 [27], and (6) RNA exosome complex [28]. Although eIF4A is not a cofactor, it is recognized by ZAP, disrupting its interaction with eIF4G and inhibiting mRNA translation [29].

It is important to examine whether ZAP also impacts host mRNA. In contrast to viral RNA, vertebrate genomes contain relatively few CpG dinucleotides [30]. This reduction in CpG sequences is primarily due to C-to-T mutations, driven by CG-specific DNA methyltransferases, which result in a lower abundance of CpG dinucleotides. Consequently, ZAP predominantly targets viral RNAs without significantly affecting host mRNA, as the latter contains fewer CpG sites for ZAP recognition [18,31].

**Table 1:** Cofactors of ZAP.

Cofactors of ZAP	Effects on Viruses	References
TRIM25	Regulating ZAP pre-mRNA splicing Enhancing ZAP binding to SINV RNA	[22,23,24]
KHNYN	Viral RNA degradation	[23]
Riplet	Enhancing degradation of viral mRNAs Co-operating with TRIM25	[24]
p72 RNA helicase (DDX17 or DEAD-box RNA helicase)	Recruiting RNA exosomes and degradation machines	[25]
DCP1A-DCP2	Inhibiting translation	[26]
XRN1	5′ to 3′ RNA degradation	[27]
PARN deadenylase	Degradation of the poly(A) tail	[32,33]
RNA exosome	3′ to 5′ RNA degradation	[5,34]
OAS3	Producing 2′-5′ oligoadenylate molecules	[20]
RNaseL	Cleaving ssRNA sequences at UpU and UpA dinucleotides sites on activation with 2′-5′ oligoadenylate	[20]

## 3. ZCCHC Family

Human CCHC-type zinc finger proteins, annotated as ZCCHC1 to ZCCHC25, contain a conserved 18-residue domain with the CX_2_CX_4_HX_4_C consensus sequence, commonly referred to as a zinc knuckle. In this sequence, “C” represents cysteine, “H” represents histidine, and “X” denotes any amino acid, with the exception of ZCCHC23, because its histidine (H) residue is substituted by asparagine (N) [35,36].

### 3.1. ZCCHC3

ZCCHC3, a CCHC-type zinc-finger-containing protein, is expressed in a variety of cell types, including epithelial cells, monocytic cells, and T-cell lines, with predominant localization in the cytoplasm [37]. ZCCHC3 has been shown to interact with retinoic-acid-inducible gene I (RIG-I) and melanoma-differentiation-associated protein 5 (MDA5), two key sensors of viral RNA [38,39]. Normally, the expression of RIG-I and MDA5 is minimal in most cells, but it significantly increases when viral RNA invades the host cell. ZCCHC3 functions as a co-receptor for these proteins, enhancing their antiviral activity [39].

ZCCHC3 directly binds to double-stranded RNA (dsRNA) and recruits RIG-I, MDA5, and E3 ubiquitin ligase TRIM25. TRIM25 activates RIG-I and MDA5 by inducing K63-linked polyubiquitination, which is essential for their antiviral signaling [40]. RIG-I and MDA5 differ in their C-terminal domains (CTDs), which are responsible for sensing distinct types of viral RNA, allowing for different RNA recognition preferences [41,42]. Despite these differences, both proteins activate similar downstream signaling pathways. Once viral RNA is recognized, RIG-I and MDA5 interact with the mitochondrial protein VISA, initiating a cascade that activates transcription factors such as IRF3 and NF-kB. These transcription factors drive the expression of antiviral genes, thereby contributing to the innate immune response [43,44,45,46].

Toll-like receptor 3 (TLR3) recognizes extracellular viral double-stranded RNA (dsRNA) and its synthetic analog poly(I:C), promoting innate immune responses through the recruitment of TRIF to TLR3, with the involvement of ZCCHC3 [47,48,49,50,51,52]. In a study involving influenza A virus (H9N2), where the viral nucleic acid is negative-sense single-stranded RNA, it was confirmed that ZCCHC3 promotes antiviral activity, evidenced by an increase in IFN-β expression along with elevated mRNA levels of related cytokines, such as IL-6 and TNF-α, in cells overexpressing ZCCHC3 compared to ZCCHC3-knockout mutants [53].

Most recently, interaction motifs between ZCCHC3 and HIV-1 single-stranded RNA (ssRNA) have been identified [37]. This study proposed two mechanisms: (1) sequestration of the HIV-1 genome into the P-body by the zinc finger (ZnF) motifs of ZCCHC3 binding directly to the long terminal repeat (LTR) of HIV-1 genomic RNA, and (2) the binding of ZCCHC3’s middle-folded domain (MF) to the HIV-1 Gag nucleocapsid (GagNC), preventing viral genome recruitment and resulting in genome-deficient virions, thereby inhibiting HIV-1 production [37].

### 3.2. ZCCHC2, ZCCHC7, and ZCCHC14

In addition to canonical poly(A) polymerases (PAPs) that synthesize mRNA poly(A) tails, vertebrates possess various noncanonical PAPs that modify RNA. One such group of noncanonical PAPs is the terminal nucleotidyltransferase 4 (TENT4), which includes TENT4A (PAPD7, TUT5, hTRF4-1, or POLS) and TENT4B (PAPD5, TUT7, hTRF4-2, or GLD4). These enzymes are involved in adding not only adenosine but also guanosine to the 3′ end of mRNA, resulting in what is known as a “mixed poly(A) tail” [54]. Unlike the canonical poly(A) tail that consists solely of adenosines, mixed poly(A) tails contain other nucleotides as well. In vitro incorporation assays have demonstrated that mixed poly(A) tailing by TENT4 primarily involves the addition of single nucleotides within longer poly(A) tails (≥25 nt). Among these nucleotides, non-adenosine bases are incorporated at the following frequencies: 15.5% for guanosine, 5.7% for uridine, and 5.2% for cytosine [55].

The addition of a single non-adenosine base at the 3′ end by TENT4A/B enhances RNA stability by preventing or slowing the degradation of mRNA by the CNOT complex [55,56,57].

TENT4 can directly target viral nucleic acids introduced into the host during infection by regulating viral RNA tailing with involvement of several ZCCHC family proteins, including ZCCHC14, ZCCHC7, and ZCCHC2, facilitating this TENT4-mediated activity. These three can be categorized into two functions: (1) enhancing the stabilization of viral RNA (ZCCHC14 and ZCCHC2) and (2) degradation of viral RNA (ZCCHC7).

#### 3.2.1. ZCCHC2 and ZCCHC14

ZCCHC14 has been shown to interact with TENT4 during infections caused by HBV, HCMV, and HAV [58,59,60]. Studies have demonstrated that HBV viral RNA undergoes more rapid degradation, with reduced viral gene expression and a shortened 3′ tail, after treatment with the dihydroquinolizinone (DHQ) compound RG7834, a drug that inhibits TENT4 activity. This inhibition leads to decreased RNA stability and viral replication [59,60]. These findings suggest that HBV RNA gains stability and delays degradation via the host TENT4-mediated tailing process [58].

A recent study found that the post-transcriptional regulatory element (PRE) in the 3′ untranslated region (UTR) of HBV mRNA contains a characteristic stem–loop structure, also known as the pentaloop or HBV stem–loop alpha (SLα), which includes a specific CNGGN sequence that ZCCHC14 recognizes and binds to this structure [61]. The study further revealed that TENT4 regulates mRNA tails through a cis-acting RNA element and proposed a mechanism for its interaction with ZCCHC14. While HBV contains this stem-loop structure near its 3′ end (PRE: 1297–1320; WPRE: 1426–1445), human cytomegalovirus (HCMV) also possesses a similar SLα-like CNGGN pentaloop in its sub-RNA2.7 genome (referred to here as SL2.7) near the 5′ end (429–451) [61]. The sterile alpha motif (SAM) domain in the central region of ZCCHC14 recognizes and binds to these stem–loop structures, subsequently recruiting TENT4, which elongates the viral RNA 3′ tail through mixed tailing, thereby enhancing viral RNA stability [61].

While TENT4’s role in extending the 3′ poly(A) tail, enhancing stability via guanylation, and promoting viral replication has been confirmed in both HBV and HCMV RNA, its mechanism in hepatitis A virus (HAV) RNA appears to differ slightly. HAV, unlike HBV or HCMV, synthesizes RNA from the positive strand to the negative strand using its own RNA-dependent RNA polymerase (3Dpol) rather than relying on the host’s polymerases [62]. Despite this distinction, the ZCCHC14–TENT4 complex also interacts with HAV RNA [63,64,65]. Similar to HCMV subRNA2.7 (SL2.7), HAV RNA contains a pentaloop structure within its 5′ UTR, particularly within the Vb stem–loop of the internal ribosome entry site (IRES), where the ZCCHC14–TENT4 complex binds [64].

The inhibition study using RG7834 treatment showed that TENT4 did not alter the 3′ end tail length of HAV RNA, suggesting that the ZCCHC14–TENT4 complex is essential for cap-independent translation initiated by HAV IRES [66]. However, another study found that the synthesis of viral RNA was significantly reduced when ZCCHC14 was knocked out, while HAV IRES’s translation-initiating activity remained unaffected [65]. This implies that the ZCCHC14–TENT4 complex plays a critical role in the late stage of the RNA replication cycle, just before protein translation [65].

There are two distinct TENT4-binding sites within ZCCHC14, located at the N-terminal (Z14-N) and C-terminal (Z14-C) regions. These sites, named D1 near the N-terminal and D4 near the C-terminal, respectively, bind with TENT4. According to this study, D4, an unstructured downstream domain, is essential for ZCCHC14’s RNA-binding activity with SAM [64]. This suggests that the D4 domain plays a role in the interaction with both RNA and TENT4, leading researchers to hypothesize that RNA binding to ZCCHC14 triggers a conformational change in the D4 domain, facilitating TENT4 binding, or vice versa [64]. In addition to ZCCHC14, ZCCHC2 also functions as an adapter protein for recruiting and interacting with TENT4 [64]. ZCCHC2 contains a PX domain, long intrinsically disordered regions, and a CCHC-type zinc finger domain [67]. While ZCCHC2 and ZCCHC14 are related, a key difference is that ZCCHC2 lacks the SAM domain required for interaction with the CNGGN pentaloop [67]. ZCCHC2’s zinc finger motif at the C-terminal binds to the K5 element, which contains a conserved RNA motif in the 3′ UTR of Aichi virus 1 (AIV-1) viral RNA [67]. This study also revealed that TENT4 is recruited to the N-terminal of ZCCHC2, regulating mixed tailing at the viral RNA’s 3′ end, which enhances RNA stability by preventing deadenylation. The K5 motif, which contains a three-hairpin structure, is critical for maintaining viral RNA stability [67].

#### 3.2.2. ZCCHC7

While ZCCHC14 forms a complex with TENT4 in the cytoplasm, ZCCHC7 (AIR1) interacts with TENT4 (specifically TENT4B) and hMTR4 in the nucleolus to form the nuclear TRAMP complex [68,69]. This complex is primarily involved in the adenylation of host rRNA degradation products [70,71]. Upon the invasion of cytoplasmic RNA viruses such as VSV, SINV, or RVFV into host cells, the proteins ZCCHC7, TENT4B, and MTR4, are exported from the nucleus to the cytoplasm via the nuclear export protein CRM1. ZCCHC7 selectively binds to viral RNA in the cytoplasm, and the helicase MTR4 unwinds the secondary structures of viral RNA, feeding it into RNA exosome complexes such as RRP6 and DIS3 for degradation [69,72,73].

ZCCHC7 binds to TENT4 via its zinc finger domain [64] and plays a role in the degradation of viral RNA, positioning it as a potential antiviral target. Research has shown viral RNA levels increase when ZCCHC7 is deleted, though further investigation is required to fully understand its mechanism and potential applications in antiviral therapy.

### 3.3. ZCCHC6 and ZCCHC11

RNA stability and degradation are regulated through several pathways. The traditional mRNA poly(A) tailing method, where RNA polymerase adds adenosines to the 3′ end of mRNA to increase RNA stability, is well known. However, with advancements in 3′ end sequencing technologies, it has been discovered that non-adenosine nucleotides can also be added to the poly(A) tail [54]. The addition of non-templated nucleotides by TENT proteins is an important mechanism controlling RNA decay. TENT polymerizing activities are classified into two types: poly(A) polymerases (PAPs), which mainly add adenosines [74], and terminal uridylyltransferases (TUTs), which add uridines [75,76].

TENT3A (TUT4, ZCCHC11, and PAPD3) and TENT3B (TUT7 and ZCCHC6) are mammalian proteins that are homologous to CDE-1 in C. elegans and Cid1 in Schizosaccharomyces pombe [77,78,79,80]. ZCCHC6 and ZCCHC11 play a key role in regulating the degradation of histone mRNA in normal cells [81] and maternal mRNA during early zygotic development [82]. However, during infection with single-stranded RNA viruses—whether positive or negative strand—ZCCHC6 and ZCCHC11 induce untemplated uridylation at the 3′ end of viral RNA, leading to antiviral effects. This mechanism involves recruiting the 3′-5′ exoribonuclease DIS3L2, which degrades viral mRNA and prevents virus replication [83].

This process mirrors how CDE-1 promotes viral RNA degradation by uridylating the 3′ end of OrV’s RNA genome in C. elegans when infected with OrV. Similarly, ZCCHC6 and ZCCHC11 in mammalian cells uridylate influenza A virus (IAV) mRNA, particularly targeting mRNA with a poly(A) tail shorter than 25 nucleotides during infection [84]. Experiments results suggest that the UU site in viral mRNA synthesized by uridylation becomes a signal for uridylation-dependent RNA decay [84], and degradation occurs via XRN1 and other RNA exosome components. This indicates that ZCCHC6 and ZCCHC11 act as an early barrier, blocking viral mRNA expression during the early stages of IAV infection [76].

Another study demonstrated that ZCCHC6 and ZCCHC11 uridylate only subgenomic transcripts with poly(A) tails shorter than 22 nucleotides during mouse embryonic fibroblast (MEF) cell infection with mouse hepatitis virus (MHV), triggering transcript decay [85]. DIS3L2 also mediates the degradation of polyuridylated mRNA, leading to decreased gene expression. However, further research is needed to clarify the precise interactions between TUTs, including ZCCHC6 and ZCCHC11, and viral RNA [84,86].

### 3.4. ZCCHC21

ZCCHC21, also known as RNA-binding motif protein 4 (RBM4) or LARK, was first identified in Drosophila. It contains a CCHC-type zinc finger motif and two consensus RNA recognition motifs (RRMs) [87]. Proteins in the RBM family, including ZCCHC21, are implicated in viral replication and antibacterial activity [88,89,90]. The key roles of ZCCHC21 in viral infection include influencing the cellular production of certain cytokines and inflammatory-response-related proteins, and directly binding to viral genomes to suppress viral replication [91,92,93,94,95].

ZCCHC21 regulates the inflammatory response by affecting the expression of genes involved in inflammation. This regulation occurs via alternative splicing patterns of regulatory factors, including transcription factors and co-activators, during inflammatory conditions such as lipopolysaccharide (LPS) stimulation or cancer [95].

ZCCHC21 activates antiviral and antibacterial responses in shrimp by inducing the expression of immune-related molecules through NF-κB and JAK-STAT pathways, which are associated with the activation of humoral immunity. They also showed that silencing ZCCHC21 (referred to here as LARK) increased shrimp susceptibility to infection with white spot syndrome virus (WSSV) through an in vivo experiment [95]. After years, a study investigating whether ZCCHC21 (referred to here as RBM4) could regulate the innate immune pathway, as well as its role in humoral immunity, has found that ZCCHC21 increased the expression of cytokines, such as *IFNB1*, *CXCL10,* and *TNFA,* in HEK293T cells transfected with ZCCHC21 expression plasmid following poly(I:C) stimulation compared to the control group [91]. Based on these findings, they concluded that ZCCHC21 inhibits viral survival by activating the host cell’s innate immune system [91].

ZCCHC21 directly binds to viral genome sequences, functioning as an antiviral factor by interfering with viral genome replication. Studies have shown that ZCCHC21 phosphorylation can be induced by cellular stress, such as exposure to arsenite. This phosphorylation triggers the subcellular relocalization of ZCCHC21 from the nucleus to the cytoplasm and stress granules (SGs) via the MKK3/6-p38 signaling pathway [93]. In a study involving the internal ribosome entry site (IRES) of encephalomyocarditis virus (EMCV), it was found that ZCCHC21 could inhibit cap-dependent translation. Conversely, ZCCHC21 activates IRES-mediated translation, likely by promoting the association of the translation initiation factor eIF4A with IRES-containing mRNAs during cell stress signaling [93]. This effect is mediated through ZCCHC21’s binding to CU-rich elements in target mRNAs [96].

ZCCHC21 also plays a regulatory role in human endogenous retroviruses (HERVs). It binds to HERV-derived RNAs and negatively regulates their expression. Loss of ZCCHC21 leads to an increased abundance of HERV transcripts and elevated expression of the HERV envelope (env) protein [92].

In recent research on Ebola virus (EBOV), ZCCHC21 was found to inhibit viral mRNA synthesis, thereby suppressing EBOV replication [91]. The EBOV genome consists of single-stranded negative-sense RNA, organized as 3′-leader-NP-VP35-VP40-GP-VP30-VP24-L-5′-trailer. The 3′-leader region contains key regulatory elements, including the replication promoter (RP) and transcription start sequence (TSS), both critical for viral replication and transcription [97]. RP consists of two key regions, PE1 (1–55 nt) and PE2 (81–128 nt), with TSS located between them [97].

The interaction between ZCCHC21 and the EBOV RNA genome occurs via ZCCHC21’s RNA recognition motif (RRM1) located in its N-terminal region (3–68 aa) and two CU-rich regions present in the 3′ leader of the EBOV RNA genome. These regions include the CUUCUU sequence in the PE1 region and the CUCCUUCU sequence in the PE2 region. The study demonstrated that even the presence of just one of these CU-rich sequences allows ZCCHC21 to bind viral RNA, suppress mRNA production, and inhibit EBOV replication. This highlights ZCCHC21 as a potential novel target for anti-EBOV therapeutic strategies [91].

## 4. Conclusions

**Figure 2:**
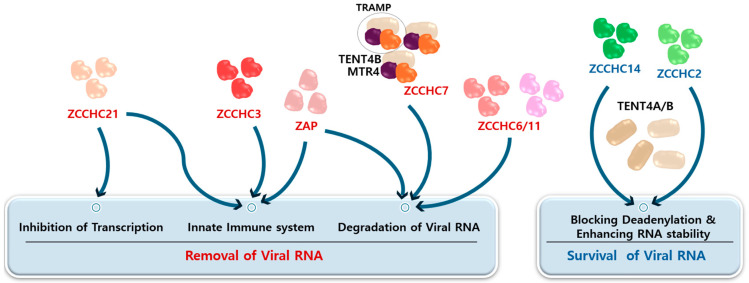
A schematic representation of the functions of viral RNA-associated host zinc finger proteins. Two major functions of zinc finger proteins are described. The first involves the degradation of viral RNA, which blocks transcription and triggers the innate immune response. The second function enhances viral stability by preventing RNA degradation. The arrows represent downstream signaling pathways involving these zinc finger proteins and their cofactors, as outlined in the corresponding sections of the text.

In this review, we have highlighted the roles of specific zinc finger proteins that regulate viral RNA and mediate antiviral signaling, identifying them as potential targets for the development of antiviral therapies. Additionally, we have summarized the roles of various zinc finger proteins and their interactions with targeted viruses, as detailed in Table 2, along with a schematic representation of their positive or negative functions for viral RNA modulation (Figure 2). This review aims to provide a foundation for future research directions focused on protecting host cells from viral infections. A major challenge in developing therapeutics based on zinc finger proteins is achieving selective modulation without disrupting host cell functions. Therefore, understanding how specific host zinc finger proteins interact with distinct motifs in viral RNA, as well as elucidating the underlying mechanisms, will significantly enhance our understanding of the fundamental cellular defense mechanisms against viral infections. The broad involvement of zinc finger proteins in innate immune responses and their selective binding to viral RNA make them strong candidates as therapeutic targets for both acute and chronic viral infections. However, more research is still necessary to translate these findings into therapeutic target development.

Moreover, antiviral therapies could have broader applications, particularly in the context of oncogenic viruses such as Epstein–Barr virus (EBV), human papillomaviruses (HPV), human T-cell lymphotropic virus type 1 (HTLV-1), human herpesvirus-8 (HHV-8), Merkel cell polyomavirus (MCPyV), and hepatitis viruses (HBV and HCV) [1]. These viruses have the potential to induce tumor formation following infection [98]. Therefore, understanding the host immune responses to these viral RNAs will not only inform strategies for combating viral infections but also provide valuable insights into the development of cancer therapies targeting virus-induced tumors. There are no antiviral therapies or vaccines that have been developed using zinc finger protein yet, but we expect the potential of CCCH-type and CCHC-type zinc finger proteins as antiviral therapy and vaccine candidates to protect human and animal populations from virus infection and to suppress cancer with the understanding of the characteristics of zinc finger protein mentioned in this review. However, further research is required for therapeutic development.

**Table 2:** Overview of zinc-finger-containing proteins and targeted viruses.

Type of Zinc Finger Protein	Name	Cellular Location	Viruses Targeted	Viral Nucleic Acid	References
CCCH-type	ZAP	Cytoplasm	Human immunodeficiency virus type 1 (HIV-1)	+ ssRNA	[15,18,19,23,32]
			Enterovirus A71 (EV-A71)	+ ssRNA	[19]
			Echovirus 7 (E7)	+ ssRNA	[20]
			Newcastle disease virus (NDV)	− ssRNA	[13]
			Influenza A virus (IAV)	− ssRNA	[13,99,100]
			Moloney murine leukaemia virus (MLV)	+ ssRNA	[7]
			Hepatitis B virus (HBV)	dsDNA	[101]
			Murid gammaherpesvirus 68 (MHV-68)	dsDNA	[102]
			Ebolavirus (EBOV) Marburg virus (MARV)	− ssRNA − ssRNA	[103]
			Sindbis virus (SINV)	+ ssRNA	[6,9,104,105]
			Semliki forest Virus (SFV)	+ ssRNA	[9,104]
			Ross River Virus (RRV) Venezuelan equine encephalitis virus	+ ssRNA + ssRNA	[104]
CCHC-type	ZCCHC3	Cytoplasm	Encephalomyocarditis virus (EMCV) Sendai virus (SeV) Vesicular stomatitis virus (VSV)	+ ssRNA − ssRNA − ssRNA	[39]
			Avian influenza virus H9N2	− ssRNA	[53]
			Herpes simplex virus 1 (HSV-1) Vaccinia virus (VACV) Murine cytomegalovirus (MCMV)	dsDNA dsDNA dsDNA	[38]
			Human immunodeficiency virus type 1 (HIV-1) Simian immunodeficiency virus (SIV) Feline immunodeficiency virus (FIV) Equine infectious anemia virus (EIAV) Murine leukemia virus (MLV)	+ ssRNA + ssRNA + ssRNA + ssRNA + ssRNA	[37]
	ZCCHC6/ ZCCHC11	Cytoplasm	Orsay virus (OrV) Influenza A virus (IAV)	+ ssRNA − ssRNA	[84]
			Mouse hepatitis virus (MHV)	+ ssRNA	[85]
	ZCCHC14	Cytoplasm	Hepatitis A virus (HAV)	+ ssRNA	[64,65,66]
			Hepatitis B virus (HBV)	dsDNA	[60,61,106,107]
			Human cytomegalovirus (HCMV)	dsDNA	[61]
	ZCCHC2	Cytoplasm	Aichi virus 1 (AiV-1)	+ ssRNA	[67]
	ZCCHC7	Nucleus Cytoplasm	Vesicular stomatitis virus (VSV) Sindbis virus (SINV) Rift Valley Fever virus (RVFV)	− ssRNA + ssRNA − ssRNA	[72]
	ZCCHC21	Nucleus Cytoplasm	Encephalomyocarditis virus (EMCV)	+ ssRNA	[93]
			Endogenous retroviruses (ERVs)	+ ssRNA	[92]
			Ebolavirus (EBOV)	− ssRNA	[91]
			White spot syndrome virus (WSSV)	dsDNA	[95]
